# Why are IPTp coverage targets so elusive in sub-Saharan Africa? A systematic review of health system barriers

**DOI:** 10.1186/1475-2875-12-353

**Published:** 2013-10-03

**Authors:** Sylla Thiam, Victoria Kimotho, Patrick Gatonga

**Affiliations:** 1 African Medical and Research Foundation (AMREF), AMREF Headquarters, P.O. Box 27691 – 00506, Nairobi, Kenya

**Keywords:** IPTp, Access, Uptake, Coverage, Barriers, Malaria, Prevention, Africa

## Abstract

**Background:**

Use of intermittent preventive treatment (IPTp) is a proven cost-effective intervention for preventing malaria in pregnancy. However, despite the roll-out of IPTp policies across Africa more than ten years ago, utilization levels remain low. This review sought to consolidate scattered evidence as to the health system barriers for IPTp coverage in the continent.

**Methods and findings:**

Relevant literature from Africa was systematically searched, reviewed and synthesized. Only studies containing primary data were considered. Studies reveal that: (i) poor leadership and governance contribute to slow decentralization of programme management, lack of harmonized guidelines, poor accountability mechanisms, such as robust monitoring and evaluation systems; (ii) low budgetary allocation towards policy implementation slows scale-up, while out-of-pocket expenditure deters women from seeking antenatal services that include IPTp; (iii) there are rampant human resource challenges including low staff motivation levels attributed to such factors as incorrect knowledge of IPTp recommendations and inadequate staffing; (iv) implementation of IPTp policies is hampered by prevailing service delivery barriers, such as long waiting time, long distances to health facilities and poor service provider/client relations; and (v) drug stock-outs and poor management of information and supply chains impair sustained availability of drugs for IPTp.

**Conclusions:**

For successful IPTp policy implementation, it is imperative that malaria control programmes target health system barriers that result in low coverage and hence programme ineffectiveness.

## Background

Each year, about 50 million women living in malaria-endemic countries throughout the world become pregnant, of whom over 50% live in high-transmission areas in Africa [1]. The disease poses serious risks to the mother, her foetus and the neonate [2]. Malaria in pregnancy is a major public health concern for which the WHO recommends a package of interventions for pregnant mothers. In areas of stable malaria transmission, recommended control measures include effective case management of malaria and anaemia, use of insecticide-treated nets and intermittent preventive treatment (IPTp).

Intermittent preventive treatment is highly cost-effective in reduction of malaria in pregnancy and consequently in reduction of neonatal mortality [3]. Reductions in neonatal mortality by up to 61.3% have been reported following IPTp administration [4]. Current IPTp guidelines stipulate that all pregnant women in areas of stable malaria transmission should receive at least two doses of intermittent preventive treatment after quickening. As the current practice of focused antenatal care recommends at least four antenatal visits, it is envisaged that a high proportion of women can receive at least two doses of IPTp. In fact, the Roll Back Malaria’s Global Action Plan target was to ensure that 80% of pregnant women received intermittent preventive therapy by 2010 [5].

However, evidence suggests elusive utilization of IPTp in Africa [6]. A recent publication in the Lancet reported that 83% of the 47 countries studied had a malaria IPT policy. In these countries, only 25% of pregnant women received at least one dose of treatment. Coverage of IPT was lowest in areas of high-intensity transmission of malaria. This phenomenon was widespread. In Malawi and Kenya, among the earliest countries to implement IPTp policy, coverage is estimated at 80.7% and 35.5% respectively. Many countries such as Somalia, Benin, Burkina Faso and Congo have coverage rates of below 10%. Madagascar and the Central Africa Republic have coverage rates of 12% and 11.8% respectively. These low coverage rates are in spite of high antenatal care attendance [7].

Why is IPTp coverage so low in Africa? Studies have shown that performance of global and national health interventions, such as IPTp, is linked to performance of health systems within which the interventions are delivered [8]. Therefore, in order to comprehensively respond to this question, it is important to consider components of the health system in order to identify IPTp scale-up barriers. Various studies have attempted to identify individual elements of the health system and the barriers related to these elements with regard to IPTp scale-up. However, a comprehensive review of these barriers is critical in order to provide consolidated evidence. By examining health system building blocks, this paper reviews existing evidence in an attempt to elucidate why achievement of optimal IPTp coverage remains elusive.

## Methods

A systematic review of literature was conducted. MEDLINE, PubMed, and Google Scholar were used to search literature published (in English) between 2005 and 2012 regarding barriers to utilization of IPTp in Africa. Search words used were: ‘barriers’, ‘IPTp’, ‘utilization’, ‘access’, ‘pregnant’, ‘women’, ‘sulfadoxine-pyrimethamine’, ‘fansidar’, ‘Africa’, ‘coverage’, ‘falciparum’, ‘SP’ and ‘health systems’.

From an initial pool of 19 papers, 12 relevant original research publications were selected by two independent reviewers based on the following criteria: original research study (containing primary data) addressing barriers to IPTp in Africa; and published between 2005 and 2012. References cited in these selected papers were also reviewed for additional evidence. Six papers were rejected because they were based on secondary data.

The WHO framework for health systems strengthening was used to analyze the main barriers related to health systems (Table 1). This analysis was conducted in the context of IPTp policy implementation.

**Table 1:** WHO framework for health systems strengthening

**Six building blocks for health systems strengthening**	**Expected outcome for IPTp implementation**
Leadership and governance	Strong political commitment backing malaria efforts, updated national policy, leadership and stewardship from national authority to lead the strategic planning process and to coordinate and align partners, financing framework
Sustainable financing and social protection	Timely availability of financial resources to implement the strategic plan from domestic and external sources to ensure that all population at risk are covered by the required interventions without bearing undue personal cost
Health workforce	Sufficient and well trained fairly distributed and productive staff to deliver interventions with highest possible quality
IPTp dugs, technology, infrastructure and logistics	Adequate procurement and supply management to ensure uninterrupted supply of cost effective medicines, commodities and tools for malaria prevention, diagnosis and treatment which are accessible to all populations at risk
Service delivery	Delivery of comprehensive package of cost effective and quality malaria interventions to those that need them, when and where they need them through health facilities and community based structures
Health information systems	Timely production, analysis, dissemination and use of reliable information. It includes confirmed malaria cases and deaths surveillance, information technology and mapping, logistic monitoring and evaluation

Source: WHO, 2010.

## Results

Findings of reviewed studies presented in this section are summarized in Table 2.

**Table 2:** HSS barriers associated with low IPTp coverage in sub-Saharan Africa

**Category of barriers**	**Countries**	**Keys barriers and evidence**
Leadership and governance	Zambia, Senegal, Malawi	Poor integration of services, poor accountability [16]
	Tanzania	Conflicting guidelines [11-13]
	Tanzania	Policy factors and slow decentralization processes for programme management [12,14,15]
Health financing	Zambia, Senegal, Malawi	Budgetary allocation [16]
	Tanzania	Out-of-pocket expenditure [13]
Human resources	Nigeria	Lack of health worker training on existing recommendations
		Incorrect knowledge [20]
	Tanzania	Under-staffing,
		Inadequate skills
		Poor motivation [13]
	Tanzania	Unfriendly supervision
		Limited training opportunities [14]
Products, infrastructure and logistics	Tanzania, Zambia	Drugs shortages, Water shortages [11,13,17]
Service delivery	Tanzania, Zambia	Long distance, long waiting time, ineffective
		Poor organization of educational services and lack of explanation to patients
		Failure to link intervention with benefits
		Lack of respect to clients [13,17]
	Nigeria	Poor quality of services [20,21]
	Tanzania¸Uganda	Inadequate time for service delivery [22,23]
	Zambia, Malawi, Senegal	Inadequate supervision of service delivery [16]
	Tanzania	Low rate of administration of IPTp by health workers at ANC [12]
Information systems	Tanzania	Weak monitoring and evaluation systems [13,14,16]

### Poor leadership and governance

Leadership is a central pillar that binds other health system components [9]. With respect to malaria control programmes, multilateral and bilateral organizations have provided global leadership, planning and organization support for African governments [10]. Numerous networks of alliances and programmes are in operation. What lacks in some countries is proper coordination and facilitation of regional networks to ensure harmonized approaches to IPTp policy implementation. Multiple IPTp guidelines are a challenge in some countries while in other areas multiple partners are involved in implementation of IPTp components of malaria programmes, and there have been reports of duplication of activities and running of parallel programmes [11]. It has been reported that low IPTp coverage levels could be attributed to unclear IPTp guidelines that lead to lost effectiveness of the IPTp strategy [12]. Evaluation of an IPTp roll-out programme in an African country revealed existence of conflicting health care worker guidelines for IPTp [13]. The national agency recommended two doses while sub-national agencies recommended other dosages. This conflict was cited as a source of confusion to frontline workers thereby hampering coverage of IPTp through a harmonized approach. Other supply side barriers that could be mitigated through effective leadership include limited dissemination of guideline booklets to health workers. Inadequate integration of IPTp into reproductive health programmes causes failure of health workers to consider IPTp as part of the antenatal care service package [13,14].

Centralized malaria control programmes are common in some parts of Africa [10]. In the Fifteenth Ordinary Session of the Assembly of the African Union, African leaders recognized the need for decentralization of malaria programmes, with IPTp components as a critical prerequisite to increased coverage and sustained programme effectiveness [15]. They reiterated the importance of effective leadership and governance in guiding utilization of resources, such as finances for malaria programming including IPTp. In Tanzania, in-depth interviews among national level malaria control officers revealed a need to address leadership constraints to IPTp policy implementation through decentralization [14].

### Inadequate and unsustainable financing

Many sub-Saharan countries rely on donor funding for IPTp supply and distribution. Countries with greater domestic budgetary allocations for IPTp and other malaria interventions have better performing IPTp programmes. Examples of these are Malawi, Senegal and Zambia [16]. The governments of these countries provide all of the funding for IPTp drugs, with central-level shortages reportedly due more to poor quantification than funding inadequacy. In fact, Zambia achieved a coverage of 62% of women receiving at least two doses of IPTp [17]. IPTp coverage for Malawi and Senegal are at 80.7% and 78.1% respectively, reflecting better performance than their sub-Saharan counterparts [7]. This was significantly attributed to increased domestic financing [16].

Patterns of inequity in access to IPTp are visible in Africa. These inequities can be linked to patterns of donor funding. Whilst the Global Fund against HIV, TB and malaria is geographically comprehensive in its funding strategy, providing funding largely in proportion to populations at risk, the disbursement patterns of other donors are more targeted and thus introduce inequity in the continent-wide pattern of funding. For example, of the 17 countries that received the US President’s Malaria Initiative (PMI) support by 2010, 14 had higher than equitable funding on malaria when compared to their neighbours [18].

Attendance of ANC clinics and IPTp access in countries is limited by lack of health insurance systems that would otherwise alleviate the problem of cash availability. Out-of-pocket payments for services are prohibitive to mothers in malaria-endemic areas. In a continent where women are burdened with domestic chores that require cash-at-hand financing, the women are faced with numerous priorities. Out-of-pocket health expenditure rates in 15 countries in East, South, West and Central Africa varied between 6% and 62.2% [19].

Further, indirect costs such as lost time and opportunity costs for acquiring IPTp also limit access of this intervention to mothers [13].

### Human resource challenges

Human resource inadequacy compounds challenges of leadership, governance and financing. Not only does the continent grapple with inadequate staffing to support IPTp, but the inequity in distribution of the available workforce continues to impact on IPTp delivery. Well qualified staff are often concentrated in urban areas leaving rural areas inadequately staffed [20]. Yet, it is in the rural areas where majority of pregnant mothers at risk of malaria in pregnancy live. Even where health workers are relatively more available, a multiplicity of pressing needs such as high HIV/AIDS prevalence render malaria prevention through IPTp administration a lesser priority.

Knowledge of recommended IPTp practices is also a challenge among health care workers. In a study on supply factors influencing IPTp coverage, it was revealed that only 14.7% of health providers had correct knowledge of all four recommendations for provision of IPTp [20]. Directly Observed Therapy (DOT) strategy was only practised by twenty two out of the 34 providers studied. The assumption that mothers would adhere to treatment instructions if left on their own was predominantly reported among health workers [20]. Other reported human resource challenges are lack of motivation, poor health care quality and client-provider relations. Supervision of peripheral health facilities is inadequate. Unfriendly supervision by facility health management teams has also been reported as a challenge among health facility staff. Further, limited opportunities for training and career development among staff dampens motivation, a factor that indirectly influences coverage of administration and coverage of IPTp. Moreover, indirect motivating factors for the health workforce have been cited as a challenge. These factors include adequate staff housing, reasonable quality of health facility infrastructure and essential material conditions such as adequate lighting in consultation rooms, functional and adequate equipment in laboratories and availability of furniture [14].

### Challenges with medical products, technology, infrastructure and logistics

Even when staff wish to deliver IPTp services free-of-charge as most policies stipulate, it is not always practical due to drug shortages that result from weak procurement systems. It is reported that up to 9% of clients miss IPTp due to drug shortages in some parts of Africa [14]. A case in point is a study in Tanzania where inadequate stocks of SP were reported to be a common problem [13]. This occurrence results from an interplay of several factors. Poor leadership and governance promotes lack of accountability and planning. This causes haphazard procurement procedures that fail to recognize systematic monitoring and evaluation of product flows and availability, causing delays in delivery and disbursement. Another factor contributing to these shortages is misuse of drugs such as sulphadoxine-pyrimethamine for clinical cases and RDT-negative malaria cases, not only in pregnant women but also in the general population [11].

Implementation of directly observed treatment strategies for IPTp is hindered by frequent water shortages at health facilities; attributing to the reported 7% and 2% coverage of DOTs at rural and urban health facilities respectively in Tanzania [14].

Quantification of required amounts of IPTp drugs is a challenge [11]. Lack of accurate consumption data, particularly at district levels adds to the challenge of quantification. Health facility catchment population estimates are often flawed due to under-reported census figures and failure to account for clients coming from other catchment areas. In fact for countries such as Zambia, areas near national borders experience inflow of clients from countries such as Mozambique and the Democratic Republic of Congo. Even when commodities are available, limited storage facilities limit the amount of stock available at any given time. Transportation of these commodities is not only expensive but also lacking in remote parts of Africa. Fuel, an indirect commodity requirement has progressively experienced a soaring cost trend [17]. Shortages of procurement and logistics staff also causes overburdening of available staff. The effect of this is slow procurement and delivery processes that compromise quality service delivery.

### Barriers to service delivery

Service delivery barriers are a critical impediment to IPTp scale-up. Long distances from health facilities limit access to health services particularly among women in far-to-reach communities [13]. This either totally hinders ANC attendance or prevents mothers from returning to health facilities for follow-up clinics. Even though reviewed studies report over 70% ANC attendance in most countries [21], poor quality of service delivery is evident. Reported bottlenecks in health service delivery are long waiting time [13], ineffective organization of educational sessions, lack of explanation to patients, failure to link the intervention with benefits and occasional lack of respect to clients [22]. Inadequate time for service delivery also hampers administration and follow up for IPTp [23]. An assessment in three countries - Zambia, Malawi and Senegal - that are regarded to have successful IPTp programmes revealed that quality assurance in health facilities through regular supervision is among attributed success factors [16].

### Weak information systems

Countries that perform relatively well in terms of effective IPTp coverage generally have better accountability systems [16]. This is ensured by regular tracking and feedback through monitoring and evaluation systems. In the continent, these systems are not always robust.

Programmes and staff at the local level require monitoring and evaluation indicators that are simple and easy to collect, yet in many countries information on IPTp is not collected as part of routine health management information systems. Instead, reported coverage figures have until now been based on Demographic Health Surveys (DHS), Multiple Cluster Indicator Surveys (MCIS), and national surveys. DHS and MCIS occur at intervals of approximately five years. Sub-Saharan Africa countries lack routine systems for data collection that are linked to regular supervision and feedback at health facility. Such feedback would have the dual benefits of informing on progress as well as incentivizing health workers to improve performance.

Core coverage and effect indicators for monitoring and evaluation of IPTp have been piloted in Nigeria, Kenya and Uganda [11]. The challenge is ensuring that these indicators are collected in a rigorous and systematic manner through routine data monitoring systems. The potential of technology is yet to be fully optimized in setting up monitoring and evaluation systems. Many countries rely on hand-written registers, the completion of which is not only time-consuming for health workers but may also be erroneous.

## Discussion

This review underscores the need to consider a health systems approach towards resolving programmatic bottlenecks and gaps for IPTp. It highlights fundamental barriers whose resolutions are likely to see improved outcomes and eventual impact of IPTp policies.

Successful IPTp scale-up will require effective leadership and governance to ensure that first, resources are allocated for IPTp and secondly, that these resources are effectively and efficiently utilized for the benefit of pregnant mothers in need of IPTp. It is this leadership that will ensure effective utilization of financial resources, human resources, commodity availability and quality service delivery. Through robust health information systems, this leadership will also ensure that systems are in place for accurate and timely monitoring and evaluation of relevant IPTp data. The exemplary performance of Zambia, Malawi and Senegal is partly attributed to effective leadership [16].

Without financial resources, implementing IPTp policies is nearly impossible. Sub-Saharan countries grapple with over-reliance on donor funds. Out-of-pocket expenditure for health deters positive health-seeking behaviour, thereby contributing to low demand for ANC services including IPTp [24]. Again, successes attained in some countries with regard to IPTp coverage are partly attributed to increased government commitment towards funding provision of IPTp drugs [16]. Sustainable financing mechanisms complement adequate human capital.

In many health facilities within sub-Saharan Africa, staff are often overworked, poorly remunerated, and thus not adequately motivated. For instance, studies examining nurses wellbeing in a sub-Saharan setting show high burnout and low job satisfaction [25]. Overworked and underpaid workers manifest burnout through depersonalization leading to a negative attitude towards their clients [26]. Hostile treatment by health workers strongly influences health services-seeking behaviour of clients. Perceptions on quality of services received at the health facility may influence IPTp utilization. Therefore, health workers need to be encouraged through supportive supervision to employ DOT strategy in order to boost adherence. Even though missed opportunities for delivery of IPTp are prevalent in Africa, perhaps one of the most overlooked determinant factors for IPTp utilization is health worker awareness. Development of simplified guidelines for health workers may resolve the problem of inconsistent messaging and poor adherence to policy guidelines experienced in some countries [13]. Guidelines for administration of IPTp stipulate the use of Directly Observed Therapy (DOT), yet in Nigeria, over three quarters of those that took SP reported that they were allowed to take the drug home [21]. This problem further compounds poor access to services and coverage. Treatment and preventive guidelines for malaria are dynamic, and continuous professional development is imperative. However it is noted that this alone cannot translate awareness to practice and, therefore, supportive supervision from credible peers, linked to feedback on performance and benchmarking with other facilities will be game changing [27]. Such an approach is likely to enforce the practice of administration of IPTp among health workers.

While service delivery provides the frontline interface between the health system and its beneficiaries, reviewed evidence reveals that this interface is fraught with challenges of inaccessibility and poor quality. Experience from successful countries demonstrates that interventions, such as community mobilization through involvement of community health workers, plays a role in promoting ANC attendance and educating on the need to prevent malaria in pregnancy [16]. Once demand for ANC services is enhanced, efficiency in service delivery may be improved through such approaches like service and programme integration and improved client-provider relations. Training on customer service and IPTp policy recommendations is necessary for health workers. Concurrently, health systems must ensure that the supply chain is effective to deliver essential commodities such as IPTp and related anti-malarial drugs. Lack of supplies at health facilities due to chronic stock outs reduces the motivation of frontline health workers to effectively deliver high impact interventions such as IPTp. Also it may reduce the use of health services by clients. These could be overcome through improved management of supply chain systems that are responsive to the demand at health facilities. Robust information management systems not only contribute to better accountability but also to responsive rather than reactive systems. They provide real-time data on demands, shortages and stocks. Staff training on the need for and set-up of functional monitoring and evaluation systems is imperative. Some countries such as Senegal have taken significant steps to improve their information systems. The country has hired new staff and established a web-based management system [16].

While this review consolidates existing evidence on health system barriers, it is recognized that health systems research specific to identifying programmatic gaps for IPTp scale-up is scanty. More studies should be undertaken in order to inform country specific programmes. In particular, it is important to explore determinants of supply side practices that limit coverage of IPTp. In fact, missed opportunities for IPT administration could be an important indicator in monitoring and evaluation systems for malaria programmes at sub-national, national and regional levels. Lastly in consideration of the evidence provided, it is noted that the scope of reviewed literature is limited by scarcity of data from across all regions of Africa. Though the cited barriers are likely to be widespread, one cannot decisively conclude so. Again, most reviewed studies are limited in the degree to which they holistically explored barriers relating to the supply and demand side of IPTp policy implementation, both quantitatively and qualitatively. Such limitations may have trickled down to this review.

## Conclusion and recommendations

Though IPTp remains an important strategy in the prevention of malaria in pregnancy, health system hurdles need to be overcome. To sustain the gains attained in IPTp implementation, and to improve coverage, intervention programmes must adopt a health system approach towards resolving barriers that hamper optimal coverage of IPTp. Furthermore, these challenges are common to most public health programmes in Africa and, therefore, health systems strengthening should be a component of all public interventions. Research and regular assessments must accompany programme implementation to gather more evidence and provide adequate guidance. Innovative approaches such as combination of interventions and enhanced integration are needed to better prevent malaria among pregnant women.

Limited individual level factors strongly associated with the use of IPTp in literature combined with some findings that adherence levels are high among women offered IPTp suggest that factors related to health-care provider behaviour are more influential in the coverage of IPTp than most measurable individual level factors. Evaluations of both health care provider practices and community perceptions and demand regarding IPTp uptake are needed to measure the extent of the problem and develop targeted interventions at improving access to IPTp.

## Competing interests

The authors declare that they have no competing interests.

## Authors’ contributions

ST conceptualized the study, designed the methodology, analysed data and provided technical review support throughout the development of the paper. VK reviewed the paper in its various stages of development while PG drafted the manuscript. All authors read and approved the final manuscript.
